# Effects of inorganic mulching on soil hydrothermal environment and tomato characters in the presence of unheated greenhouse cultivation

**DOI:** 10.1038/s41598-024-54896-y

**Published:** 2024-02-22

**Authors:** Yanyan Dai, Pengfei Zhang, Jinlong Chao, Geng Liu, Ligang Guo, Masateru Senge

**Affiliations:** 1 School of Geographic Sciences, Taiyuan Normal University, 319 Daxue Avenue Yuci District, Jinzhong, 030619 People’s Republic of China; 2 Shanxi Key Laboratory of Earth Surface Processes and Resource Ecology Security in Fenhe River Valleye, Taiyuan Normal University, 319 Daxue Avenue Yuci District, Jinzhong, 030619 People’s Republic of China; 3 School of Economics and Management, Taiyuan Normal University, 319 Daxue Avenue Yuci District, Jinzhong, 030619 People’s Republic of China; 4 Union-Infrastructure Maintenance Laboratory, Gifu University, 1-1 Yanagido, Gifu, 501-1193 Japan

**Keywords:** Inorganic mulching, Soil temperature, Soil moisture, Water use efficiency, Ecology, Plant sciences

## Abstract

The present study was conducted by cultivating tomato (*Solanum lycopersicum ‘Provence’*) using varied inorganic mulching to investigate soil hydrothermal environment and tomato characters under unheated greenhouse cultivation in the cold zone of China. A total of 6 different treatments were adopted: no mulching (control), white film mulching (white film), black film mulching (black film), the white film with hole mulching (white hole), the black film with hole mulching (black hole), and snake skin bag mulching (snake skin). Inorganic mulching treatment significantly improved soil temperature and moisture, water use efficiency, tomato yield, and reduced soil water consumption. There was no significant difference observed in the variation of daily mean soil temperature between different mulching treatments, and the variation was in the range of 1.95–2.20 °C, which was significantly lower compared with the control (3.42 °C). The daily mean soil moisture varied significantly after different mulching treatments, with the highest level achieved by snake skin (23.37%), followed by black hole (22.55%), white hole (22.08%), white film (21.48%), black film (20.12%), and control (18.78%) in descending order. According to the research results, plastic-hole mulching, which include white hole and black hole treatments, performed better in maintaining soil temperature and moisture.

## Introduction

In the cold zone of China, winter is cold and long, which makes it difficult to guarantee the high yield and quality of vegetables due to the impact of low temperatures, frost and other adverse climatic conditions. In this area, the single-slope solar greenhouse with high back wall is the main facility for vegetables farming during winter^1,2^, which can retain heat and water, adjust climatic conditions, and produce anti-season vegetables, the influence of climatic difference on agricultural production is reduced effectively. According to the physiological characteristics of crops, vegetable farmers can adjust the hydrothermal environment in the greenhouse to create the most suitable hydrothermal conditions for the growth of crops, thus improving both yield and quality. Tomato is one of the most important vegetables in China and even in the world. It is favored by people because of its high nutritional value and unique flavor and playing an important role in people's daily life^3–5^. According to FAO statistics, in 2020, the global tomato planting area was 5.05 million hm^2^, and the output was 182 million tons. In China, the tomato planting area was 1.11 million hm^2^, and the output was 64.87 million tons. The planting area of tomato facility cultivation area is 778,100 hm^2^, accounting for 57% of the total tomato area.

Plastic film mulching is widely practiced in greenhouse vegetable production during winter in the cold zone of China. As demonstrated in a large number of studies, the use of plastic film provides an effective way to reduce soil moisture evaporation^6^, improve soil structure, retaining fertilizer^7^, inhibit weed growth, and create a microsite environment suitable for crop growth^8–11^. However, it has also been revealed by some studies that long-term full film mulching can be damaging to both the soil and the plants. For example, full film mulching leads to high temperatures in the late growth season, accelerated root senescence and inhibited yield^12,13^. Full film mulching reduces the efficiency of infiltration by rainfall^14^. At the same time, the polyethylene material used for mulching film is prone to damage, and it is difficult to achieve complete recycling across a large area. The accumulation of residual film affects the soil structure as well as water and heat transportation, thus affecting crop production^15^. The type, thickness, quantity and porosity of the mulching materials determine the drying speed and soil temperature. The treatment with impermeable materials such as plastic film minimizes the evaporation from the soil surface and maintains soil temperature. However, it does not allow irrigation water and rainwater to penetrate the soil in the root zone. In contrast, plastic-hole mulching is conducive to the infiltration of irrigation water and rainwater into soil, but hinders the preservation of soil moisture and soil temperature^16^^,^^17^. In order to solve the above issues, this study designed to explore the effects of plastic-hole mulching and snake skin bag mulching on soil moisture, temperature and tomato characters.

To sum up, greenhouse cultivation is aligned with refined and sustainable agriculture, which requires proper cultivation management and techniques as a crucial link, especially during winter in the cold zone of China. Meanwhile, it is very important to select suitable mulching materials and methods to mulch the soil surface for crop growth. There are both advantages and disadvantages demonstrated by the current mainstream method of full film mulching. Hence, the objective of this study is to explore (1) the rationality of using inorganic mulching during winter in the cold zone of China and (2) the effects of different mulching methods and mulching materials (plastic mulching, plastic-hole mulching and snake skin bag mulching) on soil hydrothermal environment and tomato characters in greenhouse, thus providing a useful reference for the selection of mulching method and mulching materials.

## Materials and methods

### Experimental site

The experiment was conducted inside an earthen-wall single-slope solar greenhouse with high back wall (80 × 5.5 m, length & width) located in Yuci county, Shanxi Province, China (37°45′53″N, 112°46′44″E) from 11 Oct. 2020 to 23 Feb. 2021. In order to preserve the internal temperature during the cold season, heat preservation quilts were unrolled after sunset and rolled up after sunrise. The soil texture of the experimental site was silty loam, the soil bulk density was 1.37 g cm^−3^ in the 0–30 cm layer, and the field capacity and permanent wilting point moisture contents were 0.36 and 0.14 cm^3^ cm^−3^, respectively.

### Experimental design and treatments

**Figure 1:**
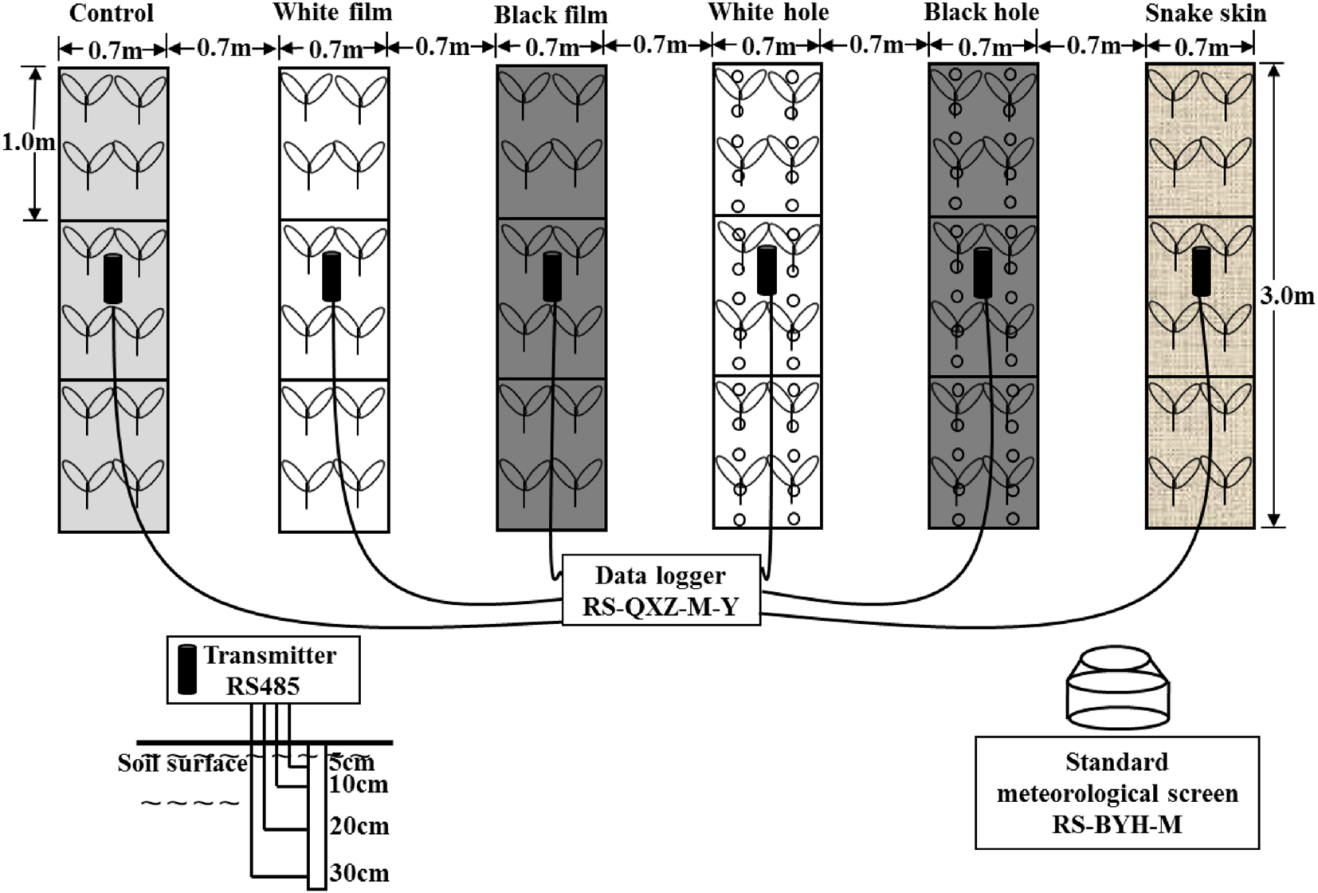
Experimental field layout and setup of various experimental devices in the greenhouse.

Six different inorganic mulching treatments were carried out during the experiment: no mulching (control), white film mulching (white film), black film mulching (black film), the white film with hole mulching (white hole), the black film with hole mulching (black hole), and snake skin bag mulching (snake skin). According to the white hole and black hole methods, the holes were cut into the membrane (white film and black film) with a diameter of 7 cm at an interval of 20 cm. Random block design was adopted for each experimental plot, and the large plot size was set to 2.1 m^2^ (3.0 × 0.7 m) for each mulching treatment with a buffer zone of 0.7 m. In order to ensure the reliability of data, each big plot was divided into three small ones (1.0 × 0.7 m). Fruit tomato (*Solanum lycopersicum ‘Provence’*) plants were taken as the experimental variety. The seedlings were cultivated at a seed company of “Taihang Pesticide Seeds” in Yuci county, Shanxi Province, China. When a height of approximately 20 cm was reached, they were transplanted to the plots as part of a randomized complete block design with 12 plants assigned to each treatment. The planting and row spacing were both 0.5 m. Lateral buds were pruned as they sprouted, and the top bud was pruned in week 7 (49–56 days after transplanting) when the plant entered the third flower-cluster stage. During the experimental period, the temperature, relative humidity, and solar radiation inside and outside the greenhouse were measured using a standard meteorological screen (RS-BYH-M, LvBo Instrument, Co. Ltd, Hangzhou, China) at an interval of 10 s (Fig. 1).

### Soil temperature and moisture measurement

Soil temperature and moisture content were measured at four different depths (5, 10, 20, and 30 cm) after each mulching treatment at an interval of 10 s by a soil temperature and moisture transmitter (RS485, LvBo Instrument, Co. Ltd) from 11 Oct. 2020 to 23 Feb. 2021. Defined as the cumulative soil–water reduction (*SWC*_*i,j*_) in the root zone (0–30 cm) between two consecutive irrigation throughout the tomato growing period, the soil water consumption (*SWC*, mm) was estimated.

1 $$SWC = \mathop \sum \limits_{i = 1}^{n} \left( {\mathop \sum \limits_{l = 1}^{m} SWC_{l,i} } \right) = \mathop \sum \limits_{i = 1}^{n} \left[ {\mathop \sum \limits_{l = 1}^{m} \left( {\theta_{IA,l,i} - \theta_{IB,l,i + 1} } \right) \times h_{l} } \right]$$

where ${\theta }_{IB,l,i}$ (cm^3^ cm^−3^) represents soil moisture content in the *l*-th soil layer just before the *i*-th irrigation,${\theta }_{IA,l,i}$ (cm^3^ cm^−3^) indicates soil moisture content of the *l*-th soil layer just after the *i*-th irrigation, ${h}_{l}$ (mm) denotes the thickness of the *l*-th soil layer, *m* refers to the number of soil layers, and *n* stands for the number of times of irrigation^18^.

The SMEP is defined as the ratio of soil moisture reduction in the *l-*th layer to the total soil moisture reduction within all the soil layers^16^. The *l-*th layer of SMEP_*l*_ was calculated as follows:

2 $$SMEP_{l} = \frac{{\theta_{l} \cdot h_{l} }}{{\mathop \sum \nolimits_{l = 1}^{n} \theta_{l} \cdot h_{l} }} \times 100$$

where ${h}_{l}$ (cm) indicates the thickness of the *l*-th soil layer and ${\theta }_{l}$ (cm^3^ cm^−3^) denotes the soil moisture content in the *l*-th soil layer.

The efficiency of irrigation water use is defined as the ratio of the amount of soil moisture used in each experimental plot to the total amount of irrigation water used during the growth of tomatoes.

### Agronomic, tomato physiological characters and fruit quality measurement

Spray irrigation (simulated rainfall) was performed randomly when plant dehydration or soil drying was observed, and the amount of irrigation water (115.16 ml, 22 times) used in each treatment was constant. Before the experiment was carried out, basal fertilizers were applied to the experimental site at rates of 160 kg hm^−2^ of nitrogen, 120 kg hm^−2^ of P_2_O_5_, and 60 kg hm^−2^ of K_2_O. Except for irrigation and fertilization, others agronomic managements (weed, pest control, pollination and so on) were the same following the local practices. Leaf chlorophyll (soil–plant analysis development: SPAD values) and leaf nitrogen (N) content (mg g^−1^) were measured weekly using a plant nutrition meter (LYS-4N, LvBo Instrument, Co. Ltd).

Red fruits were harvested once a week during the harvest season and the yield (g plant^−1^) of each plant was measured. Plant biomass was measured at the end of the experiment. The WUE was calculated according to biomass (WUE_*b*_, g mm^−1^: grams of biomass produced per mm of irrigation water), and yield (WUE_*y*_, g mm^−1^: grams of yield produced per mm of irrigation water). To assess fruit quality, twelve fully mature tomato fruits were chosen for each treatment. Besides, fruit firmness (penetrometer: GY-4, LvBo Instrument, Co. Ltd), fruit sugar content and fruit acid content (Pocket brix-acid meter: PAL-BX/ACID1, ATAGO, Co. Ltd, Tokyo, Japan) were determined, respectively. In addition, the taste index was calculated using the equation proposed by Hernández-Suárez et al.^19^ as follows:

3 $$Taste \;index = \frac{Sugar content}{{20 \times acidity}} + acidity$$

### Data analysis

Statistical analysis was performed in the form of one-way analysis of variance, the results of which were compared in the Duncan’s test against the confidence level of 5% using the R programming language.

## Results

### Meteorological characteristics

**Figure 2:**
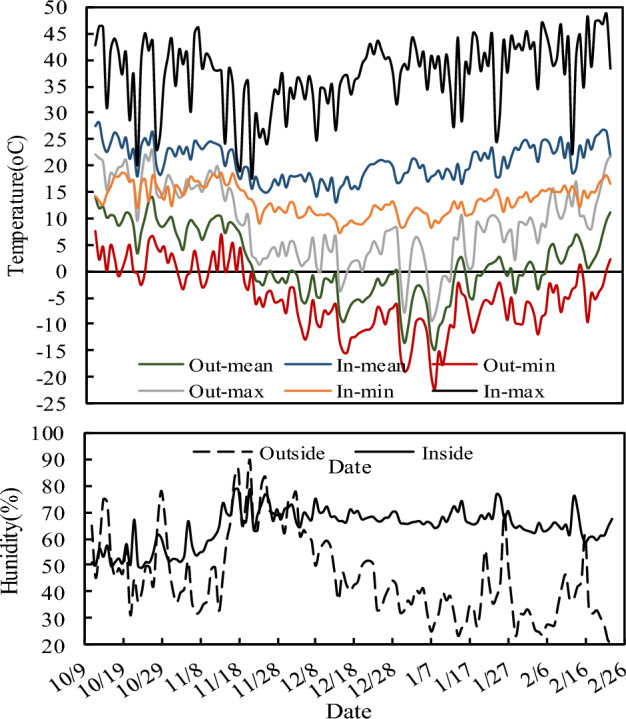
Daily temperature and humidity inside and outside the greenhouse during the growth period (11 October 2020 to 24 February 2021).

In the cold zone of China, the unheated single-slope solar greenhouse with high back wall was used to grow vegetables in winter. Temperature is known as a major factor affecting vegetable production during winter. Figure 2 shows the meteorological characteristics inside and outside the greenhouse during the growth period. Inside the greenhouse, the daily mean air temperature ranged from 13.15 to 28.30 °C, and the average temperature was 20.82 °C. The daily mean relative humidity ranged from 48.78 to 78.43%, and the average relative humidity was 64.35%. The daily mean solar radiation ranged from 1.25 to 20.52 MJ m^−2^, and the average solar radiation was 10.32 MJ m^−2^. Outside the greenhouse, the daily mean air temperature ranged from −15.03 to 14.05 °C, and the average temperature was 1.48 °C. The daily mean relative humidity ranged from 21.15 to 89.70%, and the average relative humidity was 45.91%. The daily mean solar radiation ranged from 2.86 to 28.48 MJ m^−2^, and the average solar radiation was 17.41 MJ m^−2^.

### Soil temperature and soil moisture

**Figure 3:**
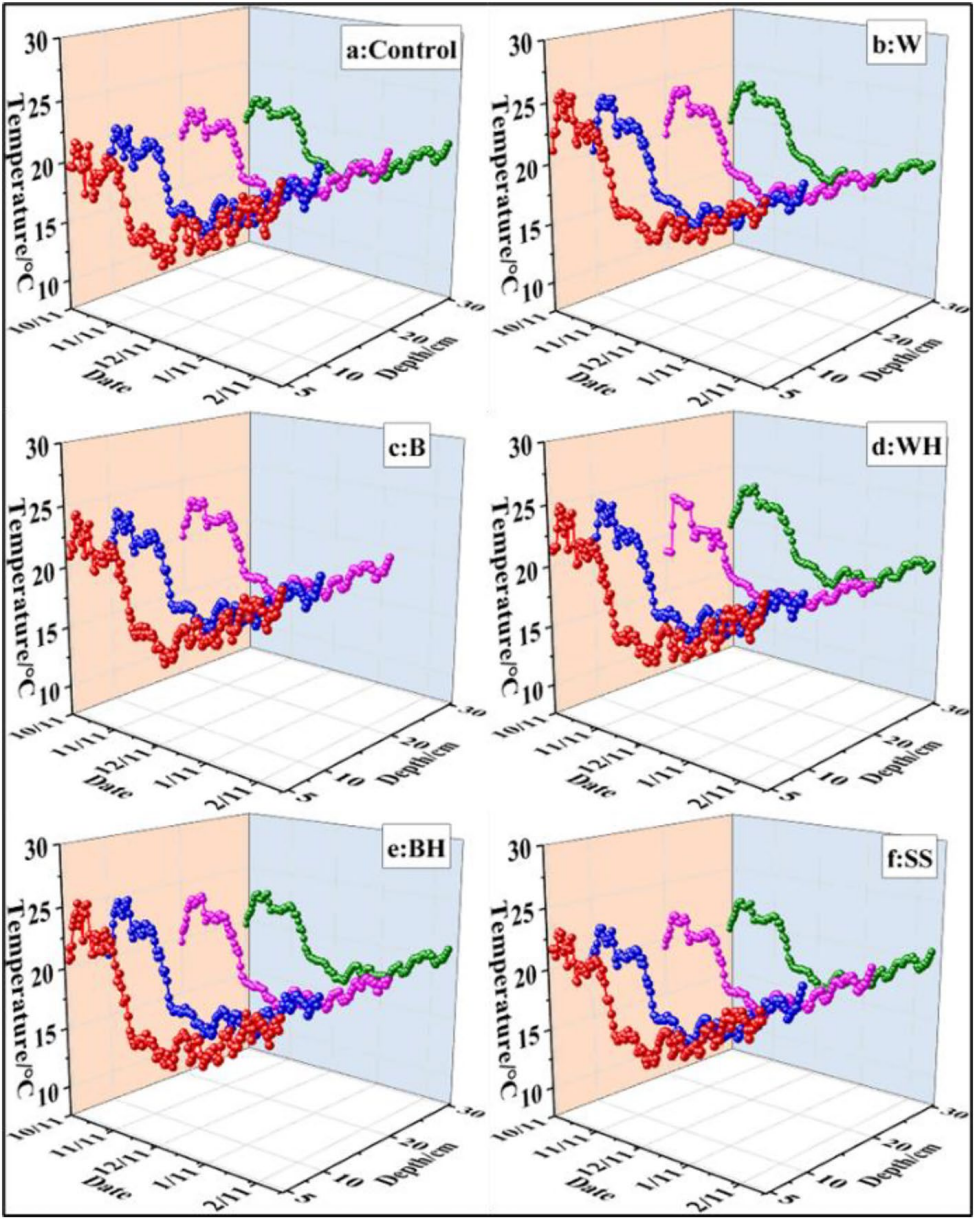
Daily mean soil temperature (°C) experimentally measured after various treatments during the growth period. *W* represents white film treatment, *B* indicates black film treatment, *W–H* denotes white hole treatment, *B–H* refers to black hole treatment, and *SS* represents snake skin treatment.

During winter, the effect of different mulching methods on soil temperature and soil moisture reached a significant extent for tomato farming in the greenhouse. According to the daily mean soil temperature (Table 1 and Fig. 3), the daily mean soil temperature of the cultivated layer (0–30 cm) covered by white film and black film during the growth period was significantly higher compared to the control and snake skin treatments. According to the daily mean soil temperature, the treatments can be divided into three categories: white film and black film treatments, where soil temperature is the highest; white hole and black hole treatments, where soil temperature is moderate; and the control and snake skin treatments, where soil temperature is the lowest. Throughout the growth period, the daily maximum soil temperature of snake skin treatment was significantly lower compared to other treatments except for white hole treatment. Also, the daily minimum temperature of the control and snake skin treatment was significantly lower compared to other treatments. There was only an insignificant difference observed in the daily soil temperature range between these five mulching treatments, ranging from 1.95 to 2.20 °C, which was significantly lower compared to the control treatment (3.42 °C). Different mulching treatments exerted significant effects on the temperature in different soil layers (5 cm, 10 cm, 20 cm and 30 cm) (Fig. 3). The control treatment showed the lowest daily mean temperature in all soil layers, while the white film and black film treatments showed the highest temperature in each soil layer. This is similar to the daily mean temperature in the whole soil layer (0–30 cm). The temperature of the white hole and black hole treatment was moderate, and that of the control and snake skin treatments was the lowest. In addition, there was no significant difference found in soil temperature between the white film and the black film treatments, as well as between the white hole and the black hole treatments.

**Table 1:** The daily mean soil temperature (DMT), daily maximum soil temperature (DMxT), daily minimum soil temperature (DMnT), daily soil temperature rang (DTR), and daily mean soil moisture (DMSM) in the 0–30 cm soil profile during the growth period after six different treatments.

Treatment	DMT (°C)		DMxT (°C)		DMnT (°C)		DTR (°C)		DMSM (%)	
Control	18.64 ± 1.9	b	20.55 ± 2.2	a	17.14 ± 1.9	b	3.42 ± 1.2	a	18.78 ± 1.9	f
W	19.39 ± 2.5	a	20.69 ± 2.8	a	18.65 ± 2.4	a	2.05 ± 0.8	b	21.48 ± 1.9	d
B	19.37 ± 2.3	a	20.62 ± 2.7	a	18.33 ± 2.2	a	2.20 ± 1.3	b	20.12 ± 1.6	e
W–H	18.82 ± 2.4	ab	20.16 ± 2.7	ab	18.16 ± 2.3	a	1.99 ± 0.9	b	22.08 ± 1.4	c
B–H	19.07 ± 2.5	ab	20.26 ± 2.5	a	18.19 ± 2.1	a	2.17 ± 0.8	b	22.55 ± 2.4	b
SS	18.60 ± 2.1	b	19.56 ± 2.4	b	17.62 ± 2.0	b	1.95 ± 0.7	b	23.37 ± 1.9	a

The data are expressed as the mean ± standard deviation. The values followed by different lowercase letters (a–d) in the same column indicate significant difference (Duncan’s test, *P* < 0.05) among the six treatments. *W* represents white film treatment, *B* indicates black film treatment, *W–H* denotes white hole treatment, *B–H* refers to black hole treatment, and *SS* represents snake skin treatment.

**Figure 4:**
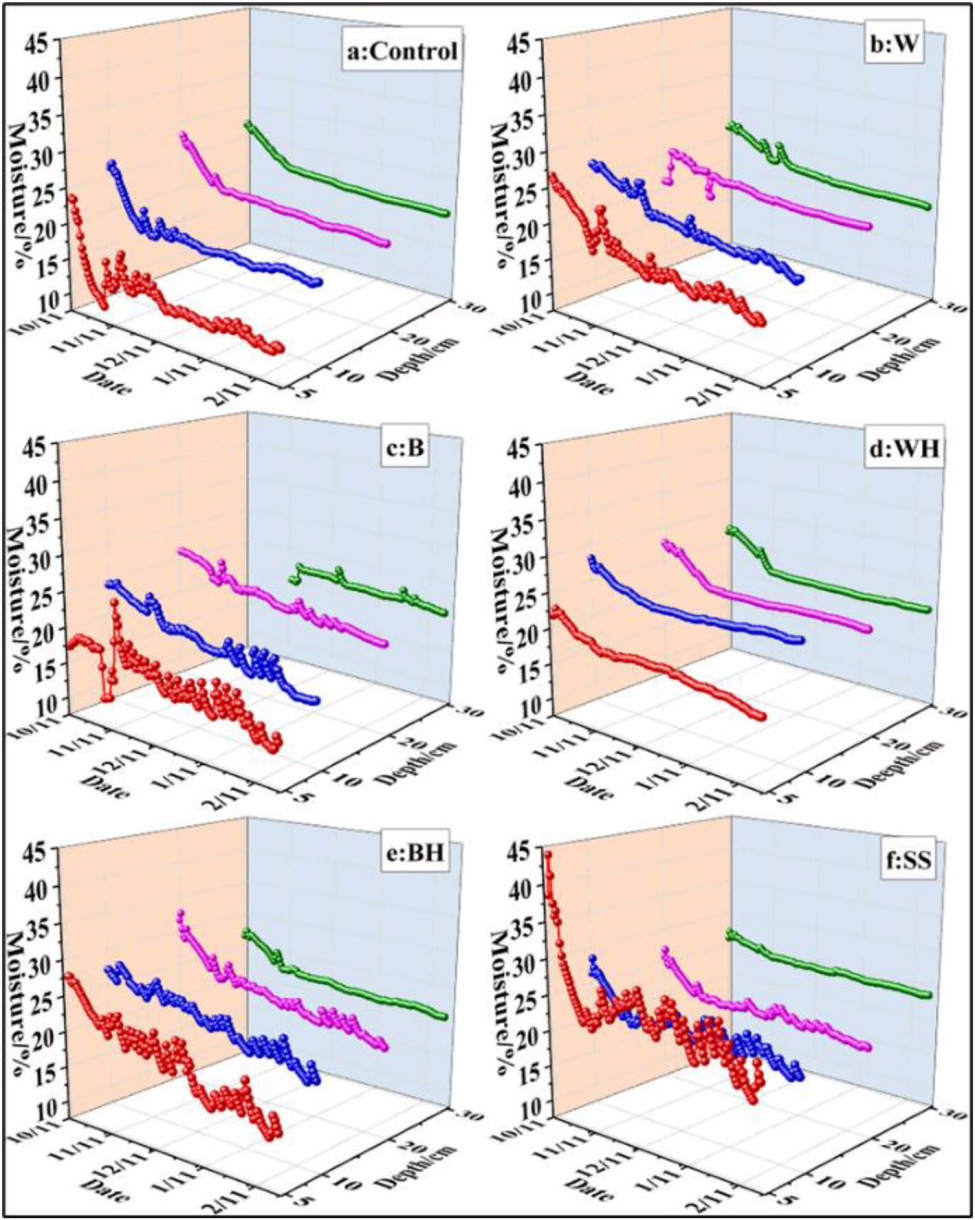
Daily mean soil moisture (%) experimentally measured after various treatments and irrigation during the growth period. *W* represents white film treatment, *B* indicates black film treatment, *W–H* denotes white hole treatment, *B–H* refers to black hole treatment, and *SS* represents snake skin treatment.

The daily mean soil moisture of the cultivated layer (0–30 cm) after different treatments made a significant difference during the growth period (Table 1), with the soil moisture in the following order: snake skin (23.37%) > black hole (22.55%) > white hole (22.08%) > white film (21.48%) > black film (20.12%) > control (18.78%). As shown in Fig. 4, within the 5 cm soil layer, the soil moisture after white film, black film, white hole, black hole and snake skin treatments was 5.51%, 3.04%, 5.59%, 6.96% and 12.06% higher compared to the control treatment, respectively. In the 10 cm soil layer, the soil moisture after white film, black film, white hole, black hole and snake skin treatments was 2.64%, 0.95%, 5.01%, 4.55% and 3.08% higher compared to the control treatment, respectively. In the 20 cm soil layer, the soil moisture after white film, black film, white hole, black hole and snake skin treatments was 1.46%, 1.19%, 1.79%, 2.59% and 1.23% higher compared to the control treatment, respectively. In the 30 cm soil layer, the soil moisture after white film, black film, white hole, black hole and snake skin treatment was 1.2%, 0.62%, 0.73%, 0.99% and 0.32% higher compared to the control treatment, respectively.

### Soil water consumption (SWC) and SMEP

**Figure 5:**
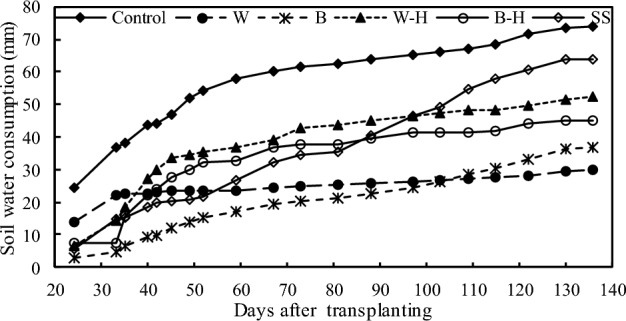
Cumulative soil water consumption in the 0–30 cm soil profile throughout the growth season after six different treatments. *W* represents white film treatment, *B* indicates black film treatment, *W–H* denotes white hole treatment, *B–H* refers to black hole treatment, and *SS* represents snake skin treatment.

The effect of different mulching treatments on SWC was significant during greenhouse tomato planting in winter, as shown in Fig. 5 and Table 2. According to the ranking of SWC from high to low, all treatments can be divided into three categories. The first one is the control treatment, with the highest SWC; the second one is the white hole, black hole and snake skin treatments, with the medium SWC; and the last one is the white film and black film treatments, with the lowest SWC. Throughout the growth period, the total SWC of each treatment was determined to be in the following order: the control (74.0 mm) > snake skin (63.7 mm) > white hole (52.4 mm) > black hole (45.1 mm) > black film (36.6 mm) > white film (29.7 mm).

**Table 2:** Effect of different mulching treatments on soil water consumption (SWC), water use efficiency for biomass (WUE_*b*_), and water use efficiency for yield (WUE_*y*_).

Treatment	SWC (mm/plant) ③		WUE_b_ (g mm−1) ➀ /③		WUE_y_ (g mm−1) ② /③	
Control	6.17 ± 2.2	a	180.26 ± 0.2	f	129.81 ± 0.3	f
W	2.47 ± 0.8	d	920.56 ± 0.3	a	640.89 ± 0.4	a
B	3.05 ± 1.5	cd	668.14 ± 0.4	b	483.47 ± 0.3	b
W–H	4.36 ± 2.3	bc	449.93 ± 0.5	d	328.09 ± 0.5	d
B-H	3.76 ± 0.8	cd	517.16 ± 0.3	c	363.28 ± 0.3	c
SS	5.31 ± 1.5	ab	376.03 ± 0.4	e	269.79 ± 0.3	e

The data are expressed as the mean ± standard deviation. The values followed by different lowercase letters (a–d) in the same column indicate significant difference (Duncan’s test, *P* < 0.05) among the six treatments. *W* represents white film treatment, *B* indicates black film treatment, *W–H* denotes white hole treatment, *B–H* refers to black hole treatment, and *SS* represents snake skin treatment.

**Figure 6:**
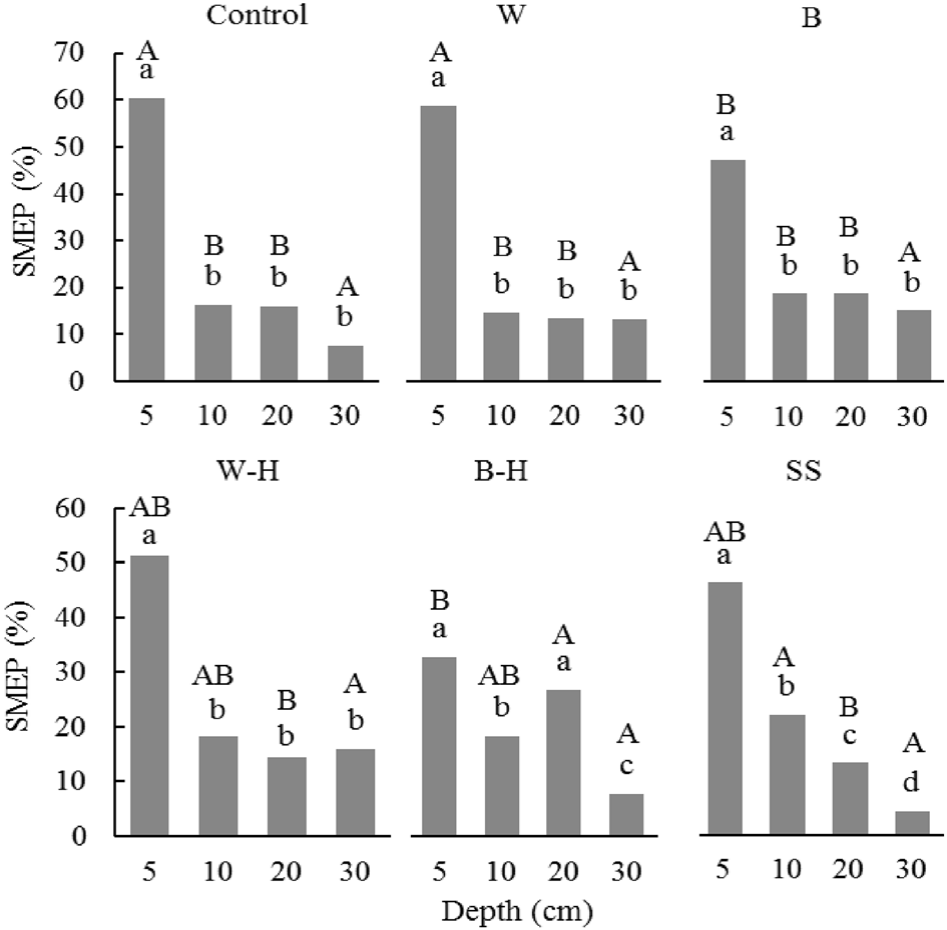
The pattern of soil moisture extraction at a depth of 5, 10, 20, and 30 cm in the 0–30 cm soil profile during the growth period. The data are expressed as the mean ± standard deviation. The lowercase letters (a–d) above the bars in the same column indicate significant difference (Duncan’s test, *P* < 0.05) at the four depths (5, 10, 20, and 30 cm) in the presence of treatment. The capital letters (A–B) above the bars in the same row indicate significant difference (Duncan’s test, *P* < 0.05) at the same depth between different treatments. *W* represents white film treatment, *B* indicates black film treatment, *W–H* denotes white hole treatment, *B–H* refers to black hole treatment, and *SS* represents snake skin treatment.

Figure 6 shows the SMEP in different soil layers during each treatment. Except for the black hole treatment, the SMEP at a 5 cm depth was significantly higher after other treatments than in other soil layers (lowercase letters). The SMEP in a soil layer with the depth of 0–10 cm after each treatment was in the following order: snake skin (79.7%) > control (76.6%) > white film (73.3%) > white hole (69.5%) > black film (66.2%) > black hole (59.7%).

### Tomato characters and fruit quality

There was no significant difference observed in tomato biomass and yield between different mulching treatments, although they were significantly higher compared to the control treatment (Table 3). There was no significant difference found in the content of leaf chlorophyll and leaf nitrogen (N) among all treatments. The WUE*s* for plant biomass and yield varied significantly between different treatments (Table 2). The WUE_*b*_ and WUE_*y*_ were found in the following order: white film > black film > black hole > white hole > snake skin > control. There was a significant difference observed in WUE*s* between these treatments, which could be divided into three categories according to the water use efficiency from high to low. The first one is the control and snake skin treatments, with the highest WUE*s*; the second one is white hole and black hole treatments, with the middle WUE*s*; and the last one is white film and black film treatments, with the lowest WUE*s*.

**Table 3:** Effect of different mulching treatments on tomato characters.

Treatment	Biomass (g) ①		Leaf chlorophyll		leaf nitrogen (mg g^−1^)		Yield (g plant^-1^) ②	
Control	1111.8 ± 396.2	b	28.4 ± 2.2	a	11.6 ± 0.7	a	800.7 ± 285.3	b
W	2278.1 ± 734.1	a	27.4 ± 3.3	a	11.3 ± 1.0	a	1586.0 ± 511.0	a
B	2036.3 ± 1032.7	a	27.5 ± 3.5	a	11.4 ± 1.1	a	1473.4 ± 747.3	a
W–H	1963.6 ± 1032.8	a	27.9 ± 2.4	a	11.5 ± 0.7	a	1431.8 ± 753.1	a
B–H	1942.6 ± 434.5	a	27.9 ± 3.1	a	11.5 ± 1.0	a	1364.6 ± 305.2	a
S	1997.4 ± 561.1	a	28.5 ± 2.2	a	11.6 ± 0.7	a	1433.1 ± 402.6	a

The data are expressed as the mean ± standard deviation. The values followed by different lowercase letters (a–c) in the same column vary significantly (*P* < 0.05) according to the Duncan’s test. *W* represents white film treatment, *B* indicates black film treatment, *W–H* denotes white hole treatment, *B–H* refers to black hole treatment, and *SS* represents snake skin treatment.

The fruit sugar content under control treatment was significantly higher than for other treatments, and white film treatment had the lowest value (Table 4). The sugar content among all treatments was in the following order: control (9.33%) > black film (8.19%) > white hole (7.73%) > snake skin (7.72%) > white film (6.88%). There was no significant difference observed in fruit acid among other treatments except for snake skin treatment. Despite the best taste index shown by the control treatment, there was no significant difference found in taste index among different mulching treatments. The fruit firmness can also be divided into three classes from high to low. The first one is the control and snake skin treatments, showing the highest fruit firmness; the second group was white film and black film treatments, showing the medium fruit firmness; and the last one is white hole and black hole treatments, showing the lowest fruit firmness.

**Table 4:** Effect of different mulching treatments on fruit quality.

Treatment	Sugar (%)		Acid (%)		Taste index		Firmness (kg)	
Control	9.33 ± 1.0	a	0.87 ± 0.17	a	1.42 ± 0.10	a	1.30 ± 0.51	ab
W	6.88 ± 0.6	c	0.83 ± 0.21	a	1.26 ± 0.13	b	1.25 ± 0.30	ab
B	8.19 ± 0.9	b	0.86 ± 0.16	a	1.35 ± 0.11	ab	1.14 ± 0.31	b
W–H	7.73 ± 0.8	b	0.93 ± 0.22	a	1.37 ± 0.14	ab	1.00 ± 0.23	b
B-H	7.63 ± 0.7	b	0.87 ± 0.18	a	1.32 ± 0.09	ab	1.00 ± 0.25	b
S	7.72 ± 1.1	b	0.64 ± 0.23	b	1.29 ± 0.11	b	1.57 ± 0.60	a

The data are expressed as the mean ± standard deviation. The values followed by different lowercase letters (a–c) in the same column vary significantly (*P* < 0.05) according to the Duncan’s test. *W* represents white film treatment, *B* indicates black film treatment, *W–H* denotes white hole treatment, *B–H* refers to black hole treatment, and *SS* represents snake skin treatment.

### Ethical approval

This study complied with the IUCN Policy Statement on Research Involving Species at Risk of Extinction and the Convention on the Trade in Endangered Species of Wild Fauna and Flora.

## Discussion

### Effects of different mulching treatments on soil hydrothermal environment

Soil moisture and temperature are two major factors affecting crop growth, and soil hydrothermal conditions can be improved by film mulching^20,21^. Film mulching can lead to a significant increase in soil temperature, thus forming a relatively closed system in the soil. This is conducive to the effective regulation of soil heat exchange^22^. In this study, it was found out that the daily mean soil temperature in the cultivated layer increased by 0.73–0.75 °C due to the plastic mulching (white film, black film) in the unheated single-slope solar greenhouse with high back wall during winter, and by 0.18–0.43 °C due to the plastic-hole mulching (white hole, black hole). By contrast, snake skin mulching made no significant difference to the soil temperature. The daily mean maximum temperature after each mulching treatment was lower than after the control treatment (except for plastic mulching), the minimum temperature was higher compared to the control treatment, and the daily soil temperature range was significantly lower compared to the control treatment. From above, it can be seen that the soil temperature after inorganic mulching treatment is higher than without mulching treatment, which is attributed to the fact that solar radiation can reach the surface through plastic film. The use of plastic film weakens long-wave back radiation under the film, thus increasing surface temperature. In addition, the water droplets on the lower surface of plastic film could decrease the longwave radiation exchange, reducing the radiation loss from soil at night and slowing down the soil temperature reduction. Another reason is the low sensible heat exchange due to the much lower air heat conductivity between the film and the soil than the turbulent diffusion on bare soil^23^^,^^24^. The temperature after plastic mulching is higher than the plastic-hole mulching, which may result from the difference in area of plastic film mulching. Plastic-hole mulching makes the soil temperature uneven. The temperature of the film-covered part is high, while the temperature of the hole part is low. Thus, in addition to the heat conduction between the soil of the hole part and the atmosphere, it also increases the heat conduction between the high-temperature soil and the low-temperature soil. Snake skin mulching obstructed the long-wave radiation to the soil and alleviated the rapid rise of soil temperature. However, due to its porosity, snake skin mulching failed to maintain the soil temperature. Thus, compared with the control treatment, it had no significant effect on the mean soil temperature during the study period, but significantly reduced the maximum soil temperature.

**Figure 7:**
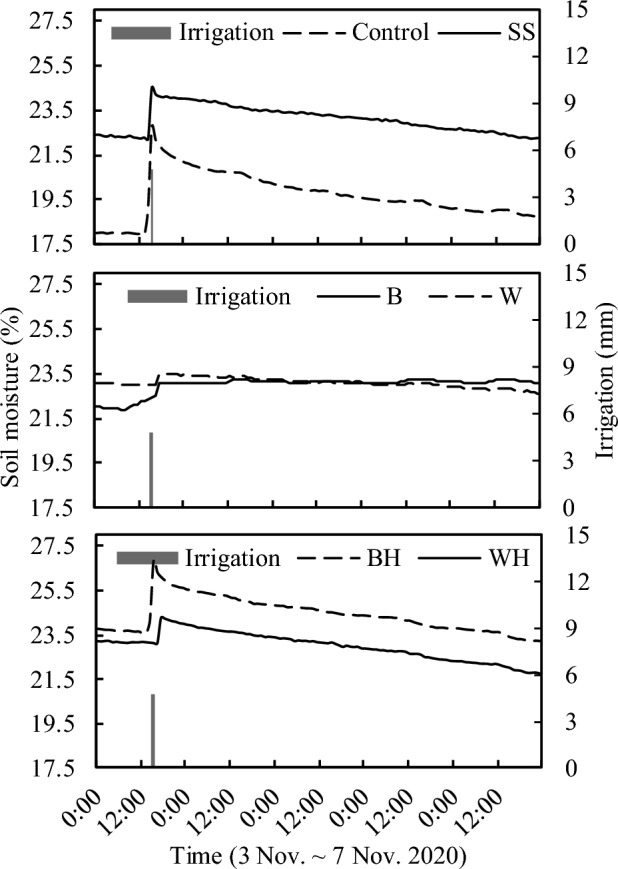
The dynamic of hourly mean soil moisture among the treatments in the earlier growth stages (3–7 November 2020) after irrigation.

Film mulching can reduce the vertical evaporation of soil moisture, thus affecting the field microclimate^25^^,^^26^. In this study, it was found out that the highest soil moisture after snake skin mulching in the 0–30 cm soil layer resulted probably from the porosity of the snake skins mulching. After irrigation, it allows water to penetrate the soil surface quickly and then seep down. However, in the process of evaporation, the snake skin mulching also blocks the long-wave radiation to the soil surface, thus significantly reducing the evaporation of the soil surface. In addition, the small water droplets, formed by the evaporating water vapor in the lower layer of the snake skin mulching, return to the soil surface due to the gravity for secondary soil infiltration, thus further increasing the soil moisture. Similarly, in the early stage of irrigation, plastic-hole mulching treatment makes the water contact the soil surface quickly through holes and diffuse. However, in the evaporation process, the film-covered part evaporates slowly and the hole part evaporates faster, thus causing soil water conduction in the soil. The above hypothesis is verified in Fig. 7, which shows that the soil moisture after control treatment, plastic-hole treatment and snake skin treatment increased rapidly, and then decreased rapidly in control treatment. Differently, it decreased slowly after plastic-hole treatment and snake skin treatment. However, the change of soil moisture after plastic treatment was insignificant both at the beginning and after irrigation.

### Effects of different mulching treatments on physiological characters and fruit quality

As an important measure taken to increase crop yield and income, plastic mulching can improve the efficiency of water and fertilizer utilization^27^. In this study, it was shown that compared with the control treatment, various inorganic mulching treatments, such as plastic mulching, plastic-hole mulching and snake skin mulching, increased biomass, WUE_*b*_ and WUE_*y*_ and tomato yield. This is consistent with the study of Deng et al.^28^ and Yan et al.^29^. Compared with open-field cultivation, mulching cultivation significantly improved crop yield, due to the effect of film warming, moisturization and improved soil structure. Thus, tomato grew vigorously in the early period, with more dry matter accumulated, which promoted the later growth of crops and boosted crop yield^30^. To be specific, the biomass, WUE*s* and tomato yield of plastic mulching were invariably higher compared to plastic-hole mulching, indicating that both black film and white film could reduce water consumption and improve yield. Additionally, the effect of white film was more significant than that of black film. As demonstrated by Zhang^31^^,^ white film improved crop yield relative to black film. Compared with the control treatment, inorganic mulching treatment significantly reduced the sugar content and taste index of tomato fruits. This is because unlike mulching treatment, the control treatment led to water deficit in the root during growth, especially at the stage of maturity. The water deficit in the root reduced the water content inside the fruit, which increased the concentration of photosynthate in the fruit, promoted the accumulation of assimilates, and thus improved the fruit quality.

## Conclusion

In general, inorganic mulching is conducive to improving soil temperature, soil moisture, WUE, biomass and tomato yield, which is essential for greenhouse cultivation during winter in the cold zone of China. In this study, the color of mulching film had no significant effect on soil temperature and humidity and tomato characters. Plastic mulching (white film and black film) significantly improved soil temperature, but not as well as plastic-hole mulching and snake skin mulching in soil moisture retention. In contrast, plastic-hole mulching (white hole, black hole) performed better in maintaining soil temperature and moisture. A reasonable design of pore size and density can improve crop yield and water use efficiency. Snake skin mulching made little difference to soil temperature, but improved soil moisture well, which makes it suitable for warm and arid climate areas.

## Data Availability

All data generated or analyzed during this study are included in this published article.
